# Anxiolytic Effect of Increased NREM Sleep after Acute Social Defeat Stress in Mice

**DOI:** 10.1007/s12264-020-00473-y

**Published:** 2020-02-24

**Authors:** Xiang Feng, Hui-Ying Zhao, Yu-Jin Shao, Hui-Fang Lou, Li-Ya Zhu, Shumin Duan, Yan-Qin Yu

**Affiliations:** Department of Neurobiology and Department of Neurology of The Second Affiliated Hospital, Zhejiang University School of Medicine, Hangzhou, 310058 China

**Keywords:** Social defeat stress, Sleep, Sleep deprivation, Anxiety, Anxiolytic effect

## Abstract

Social defeat stress (SDS) plays a major role in the pathogenesis of psychiatric disorders like anxiety and depression. Sleep is generally considered to involve recovery of the brain from prior experience during wakefulness and is altered after acute SDS. However, the effect of acute SDS on sleep/wake behavior in mice varies between studies. In addition, whether sleep changes in response to stress contribute to anxiety is not well established. Here, we first investigated the effects of acute SDS on sleep/wake states in the active period in mice. Our results showed that total sleep time (time in rapid eye-movement [REM] and non-REM [NREM] sleep) increased in the active period after acute SDS. NREM sleep increased mainly during the first 3 h after SDS, while REM sleep increased at a later time. Then, we demonstrated that the increased NREM sleep had an anxiolytic benefit in acute SDS. Mice deprived of sleep for 1 h or 3 h after acute SDS remained in a highly anxious state, while in mice with *ad libitum* sleep the anxiety rapidly faded away. Altogether, our findings suggest an anxiolytic effect of NREM sleep, and indicate a potential therapeutic strategy for anxiety.

## Introduction

Frustration in social interactions is a common problem, most often illustrated by bullying in schools. Social defeat is a major source of stress in humans and plays an important role in the pathogenesis of mental disorders such as anxiety and depression [1]. At the same time, occasional emotional reactions, including stress responses, are generally viewed as normal psychological and physical reactions to negative situations in daily life and help to cope with a frequently changing and challenging environment. However, frequent emotional reactions referred to as chronic stress are pathological conditions that increase the risk of depression and anxiety [2–6]. Therefore, timely alleviation of acute stress can prevent it from developing into a pathological emotional state.

In addition to causing affective disorders, acute stress promotes wakefulness and enhances arousal while the stressor persists [7]. Social stress also leads to sleep disturbances [7–10]. Interestingly, in contrast to the well-studied effect of stress on anxiety-like behaviors, the consequences of social defeat stress (SDS) on sleep are not clear [10–14]. An acute effect of SDS on sleep has been reported in different studies using different SDS procedures and standards of comparison. For example, the results of investigating whether wakefulness or sleep time is increased in the first 3 h after SDS are contradictory in two recently-published studies [11, 15]. On the other hand, sleep disturbances are recognized as symptomatic of anxiety disorders [16–18]. Sleep loss is anxiogenic in mice and humans [18, 19]. Previous studies have shown a sharp increase in slow-wave activity during non-rapid eye movement (NREM) sleep after a single social defeat, indicating that NREM sleep may function to cope with stress [8, 9]. Altogether, it is of interest to determine how natural sleep affects anxiety after SDS in mice, so it is necessary to analyze sleep performance after acute stress and its role in the modulation of emotion.

Rodent models of SDS are based on a resident-intruder paradigm where the intruder mouse is defeated by an aggressive resident CD-1 mouse, and they have been shown to exhibit behavioral changes [20–22] and sleep alterations [15, 20, 23]. In the present study, using an acute SDS model, we investigated the changes in sleep after acute SDS. We then examined the role of increased NREM sleep after acute SDS in anxiety regulation, and revealed a relationship between sleep alteration and anxiety.

## Materials and Methods

### Animals

Male C57BL/6J mice 8–10 weeks old were maintained under standard housing conditions on corn cob litter in an animal room with controlled temperature (22 ± 1°C) and humidity (55% ± 5%) on a 12-h light/dark cycle (lights on from 07:00 to 19:00; 07:00, zeitgeber time 0) with *ad libitum* food and water. All experiments were conducted in accordance with the Guidelines for the Care and Use of Laboratory Animals of Zhejiang University and all protocols were approved by the Institutional Animal Care Committee (ZJU2015-015-01).

### Stereotaxic Surgery for Placement of Electroencephalogram (EEG) and Electromyogram (EMG) Electrodes

Each mouse was anesthetized with ketamine and xylazine (100 mg/kg body weight and 10 mg/kg body weight, respectively, i.p. injection) and placed in a stereotaxic instrument (RWD Life Science, Shenzhen, China). The mice used for sleep recording were implanted with a custom-made EEG and EMG unit placed on the rear of the skull. EEG signals were recorded from electrodes on the frontal cortices (AP, 2 mm; ML, 1 mm). Two stainless-steel wires were inserted into neck muscles as EMG electrodes. The EEG electrodes and two stainless-steel screws for anchorage were fixed to the skull with dental cement. Tissue glue (3M Vetbond, St. Paul, MN) was used to help heal wounds and fix the EEG-EMG electrodes. Mice were allowed to recover for 1 week and briefly handled for 5 min daily for habituation.

### Acute Social Defeat Stress

Male CD-1 retired breeder mice 6–8 months old were purchased from Charles River Co. (Beijing, China) and housed singly with 3 days of screening to select those meeting the social defeat criterion [23]. Unsuitable mice that showed little aggression were excluded. Only mice showing aggression within 1 min and persistent aggressive intention were used in social defeat sessions. An entire social defeat session lasted for 10 min with ~10–15 conflicts for each intruder mouse. In cases when the intruder mouse was bleeding or had visible wounds, the intruder and corresponding CD-1 mouse were excluded. All acute social defeat sessions were carried out during the first hour of the active period. Each intruder mouse was then returned to its home cage for *ad libitum* sleep or sleep deprivation.

### Recording and Analyses of EEG and EMG

After 1 week of recovery in single housing, mice were transferred to a recording cage in a sound-attenuated box and habituated to the recording cables for 3 days. To allow free movement in the recording cage without tangling, a slip-ring device (CFS-22, Biotex, Japan) was used for the cable. The EEG and EMG signals were amplified and filtered (EEG, 0.5 Hz–100 Hz; EMG, 10 Hz–500 Hz) by an AC amplifier (Model 1700, A-M Systems, Carlsborg, WA), digitized at 200 Hz (PowerLab ML795, AD Instruments, Dunedin, Australia), and recorded by LabChart software (AD Instruments).

The sleep analysis software SleepSign (Kissei Comtec, Matsumoto, Japan) was used for sleep state scoring. All scoring was automatic on the basis of the EEG and EMG waveforms in each 4-s epoch. Sleep state was defined as follows: wakefulness, desynchronized EEG and high EMG activity; NREM sleep, synchronized EEG with high power at delta frequencies (0.5 Hz–4 Hz) and low EMG activity; REM sleep, desynchronized EEG with high power at theta frequencies (4 Hz–10 Hz) and low EMG activity. All brain state classification was validated manually.

### EEG Power Spectral Analysis

The EEG power spectral density was analyzed with NeuroExplorer (Nex Technology, Littleton, MA). To quantify the sleep quality, we used the EEG power represented by the power spectral density in the different frequency ranges relative to the average value of total power in the same epoch.

### Sleep Deprivation Procedure

Sleep deprivation was achieved through gentle touch with a soft brush pen over a period of 1 h or 3 h after acute SDS. Mice were singly housed and gently touched for 1 s–2 s as soon as they displayed signs of drowsiness.

### Behavioral Testing

#### Open Field Test (OFT)

The OFT was used to assess anxiety-related behavior and locomotor activity in an open field arena (50 × 50 × 60 cm^3^) under low light (~20 lux in the center and ~5 lux in the corners) to minimize anxiety effects. Mice were placed in the center of the arena, then the total locomotor activity in 5 min and the time in the center area were recorded and analyzed using the ANY-maze system (Stoelting, Wood Dale, IL).

#### Elevated Plus-maze (*EPM)*

The EPM consisted of a plus-shaped platform with 4 intersecting arms: 2 opposing open arms and 2 closed arms. The illuminance was controlled at ~20 lux in the open arm and ~5 lux in the closed arm. Each animal was placed in the center of the apparatus facing a closed arm and allowed to freely explore the maze for 5 min. Behavioral data from an animal that fell off the open arm was excluded from analyses. Time in the open arm was recorded using the ANY-maze system.

### Statistical Analysis

Data are presented as the mean ± SEM and were analyzed using Prism 8 (Graphpad Software, San Diego, CA). In all cases, *P* < 0.05 was considered significantly different. Multiple *t*-tests were used to analyze the sleep/wake profile and EEG power. The paired two-tailed Student’s *t*-test was used for statistical comparisons between paired groups. Two-way ANOVA followed by Tukey’s multiple comparisons was used to compare anxiety-like behaviors changing with time in 2 groups with different treatments. Associations between latency to sleep onset and behavioral measures of anxiety were tested using Pearson’s correlation.

## Results

### Acute SDS Increases Sleep in the Subsequent Active Period

Animals suffer from social defeat mainly in the active period. However, all previous studies of mice started the social defeat session in the inactive period when the duration of baseline NREM sleep is relatively longer [11–13]. In reality, stressful events are more likely to occur in the active period. Thus, the effects of stress on sleep in the active period in mice remain unclear.

**Fig. 1:**
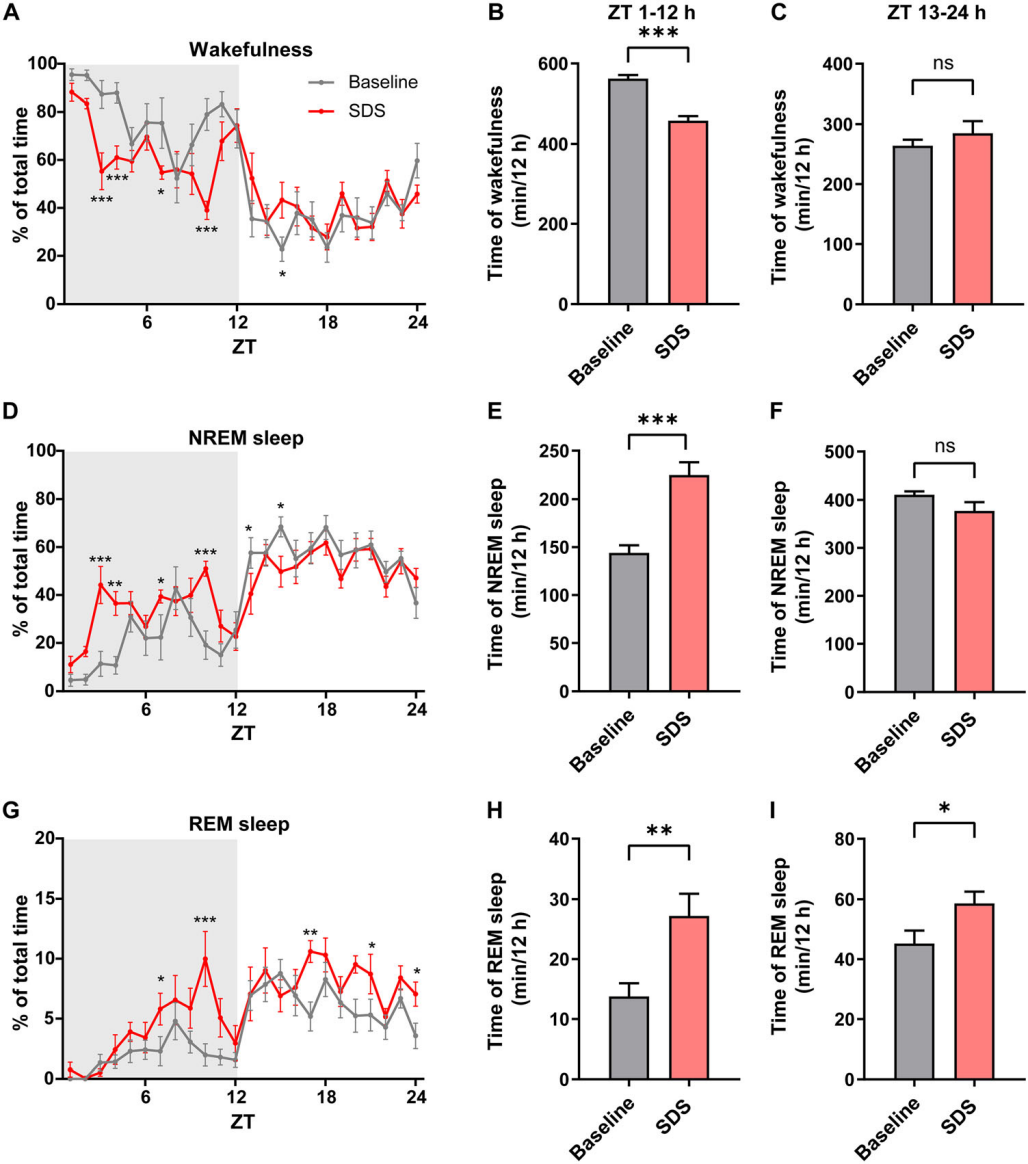
Acute social defeat stress increases NREM sleep in the active period. **A, D, G** Percentages of time spent in wakefulness (**A**), NREM sleep (**D**), and REM sleep (**G**) during 24 h following acute SDS. Acute SDS occurred at the beginning of the active period (ZT0) (shaded area indicates the active period) (*P < 0.05, **P < 0.01, ***P < 0.001 *vs* baseline, multiple *t*-test). **B, E, H** Total time in wakefulness, NREM sleep, and REM sleep for 12 h in the active period (**P < 0.01, ***P < 0.001, paired two-tailed Student’s *t*-test). **C, F, I** Total time in wakefulness, NREM sleep, and REM sleep for 12 h in the inactive period (*P < 0.05, paired two-tailed Student’s *t*-test). *n* = 7–8 mice/group; data are presented as the mean ± SEM; ns, not significant; ZT, Zeitgeber time.

First, to investigate the effects on sleep of SDS starting in the active period, we analyzed EEG and EMG recordings at baseline (the day before social defeat) and within 24 h after SDS. When returned to the home cage, the intruder mice showed a robust decrease of time in wakefulness (Fig. 1A). We compared the total amount of wakefulness within the whole 12 h in the active period or inactive period between baseline and SDS, and found that the decrease of wakefulness mainly occurred in the first 12 h of the active period (Fig. 1B). Acute SDS in the active period had a limited influence on the total time of wakefulness in the later 12 h in the inactive period (Fig. 1C). Accordingly, NREM and REM sleep increased during the corresponding periods (Fig. 1D–I). Significant increases in NREM sleep occurred at 3 h, 4 h, 7 h, 10 h, 13 h, and 15 h after SDS (Fig. 1D). Total analysis showed that NREM sleep increased in the active period but not in the subsequent inactive period (Fig. 1E, F). An increase in REM sleep only occurred at 7 h, 10 h, 17 h, 21 h, and 24 h after SDS (Fig. 1G), and the total amount of REM sleep increased in both the active (Fig. 1H) and inactive periods (Fig. 1I). These results demonstrated that acute SDS induces a decrease in wakefulness and an increase in NREM sleep in the active period and an increase in REM sleep in both the active and inactive periods. The increase in NREM sleep occurred during a limited time window in the first 3 h after SDS in the active period and was attenuated in the inactive period.

**Fig. 2:**
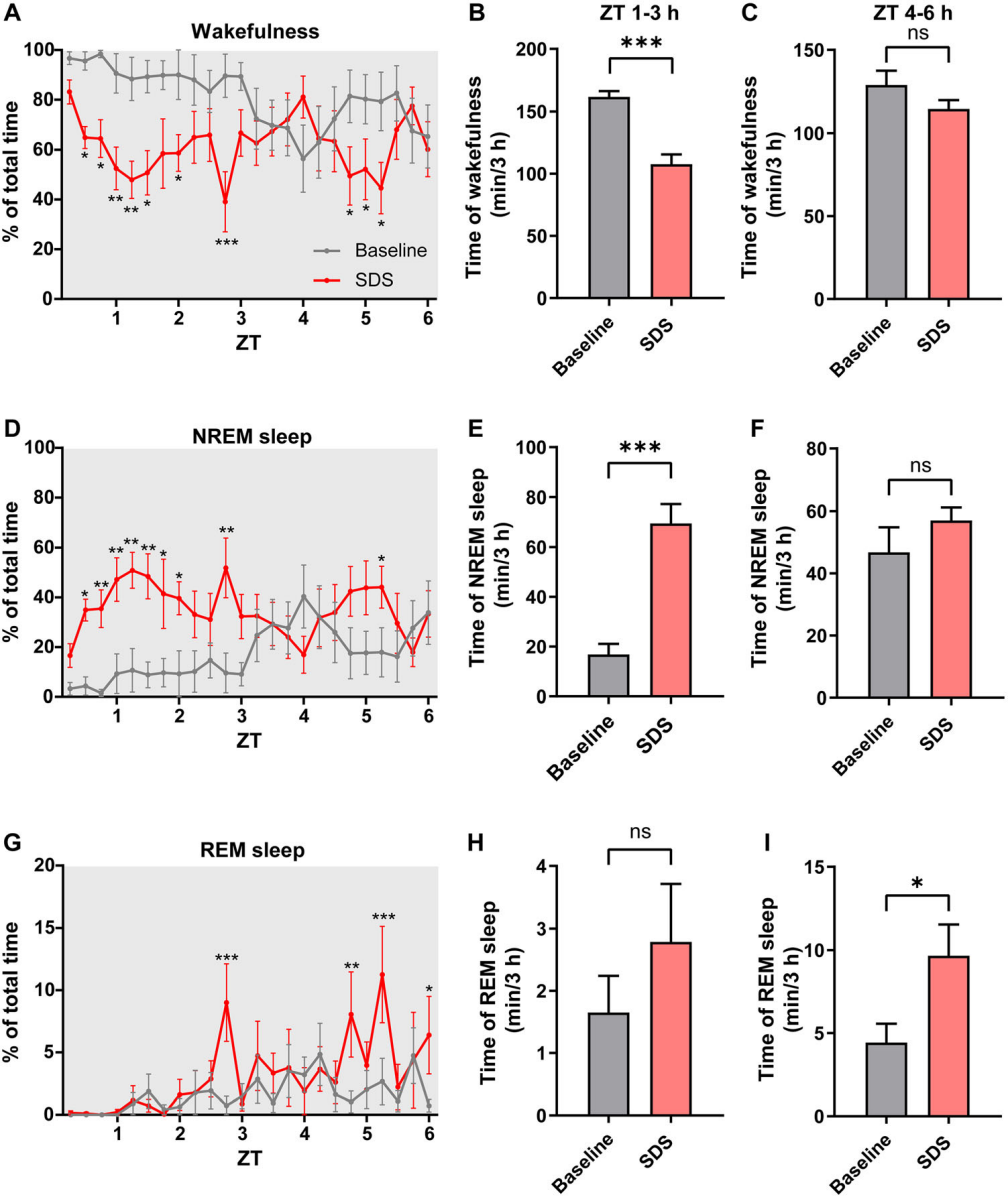
NREM sleep increases primarily within 3 h after SDS. **A, D, G** Percentages of time spent in wakefulness (**A**), NREM sleep (**D**), and REM sleep (**G**) during 6 h following acute SDS (*P < 0.05, **P < 0.01, ***P < 0.001 *vs* baseline, multiple *t*-tests). **B, E, H** Total time in wakefulness, NREM sleep, and REM sleep for 3 h from ZT1 to ZT3 (***P < 0.001, paired two-tailed Student’s *t*-test). **C, F, I** Total time in wakefulness, NREM sleep, and REM sleep for 3 h from ZT4 to ZT6 (ns, not significant, paired two-tailed Student’s *t*-test). *n* = 7–8 mice/group; data are presented as the mean ± SEM.

Next, to define the precise time window of the increased NREM sleep, we analyzed the sleep/wake profile at 15-min resolution during the first 6 h after SDS (Fig. 2A, D, G). We found that only during the first 3 h (ZT1–3 h) after SDS did the amount of NREM sleep significantly increase, while the amount of REM sleep did not change (*P* = 0.19, Fig. 2E, H). Interestingly, contrary to NREM sleep, REM sleep increased mainly in the later 3 h (ZT4–6 h), showing a delayed effect of stress (Fig. 2G–I). The strong increase in sleep after social defeat indicates that sleep may function to offset the mental load imposed on the nervous system during wakefulness.

**Fig. 3:**
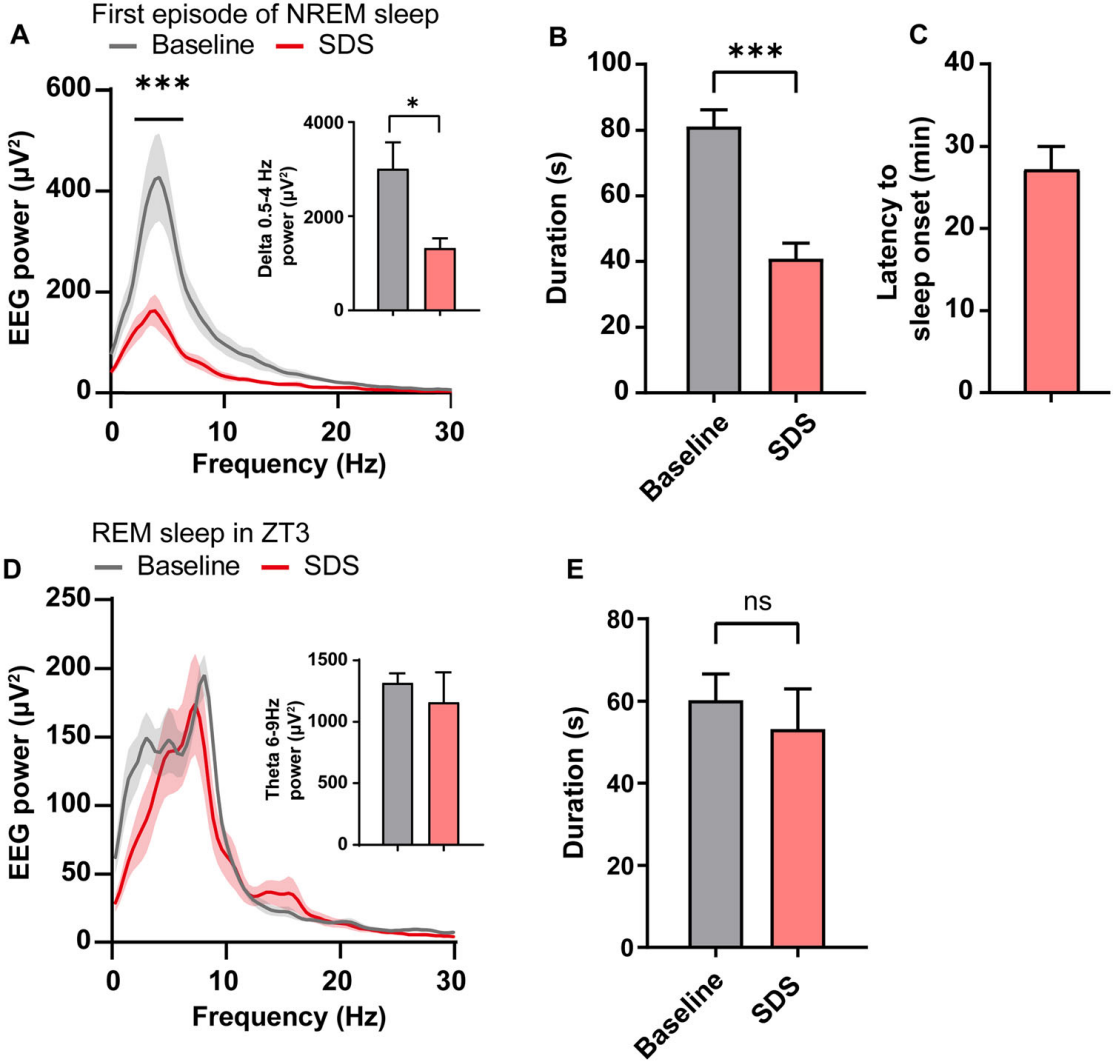
Characteristics of the first NREM sleep episode following acute SDS. **A** EEG power of baseline sleep and the first NREM sleep episode after SDS (***P < 0.001 *vs* baseline, multiple *t*-tests corrected by Bonferroni–Dunn multiple comparisons). Inset: total EEG power in the delta band (0.5 Hz–4 Hz, *P < 0.05, paired two-tailed Student’s *t*-test). **B** Duration of the first episode of NREM sleep compared with baseline sleep (***P < 0.001, paired two-tailed Student’s *t*-test). **C** Latency to the first NREM sleep episode after SDS. **D** EEG power of natural REM sleep and the first REM sleep episode after SDS (multiple *t*-tests corrected by Bonferroni–Dunn multiple comparisons). Inset: total EEG power in the theta band (6 Hz–9 Hz, *P* > 0.05, paired two-tailed Student’s *t*-test). **E** Duration of the first episode of REM sleep compared with baseline sleep. *n* = 8–10 mice/group.

Moreover, we compared the first episode of NREM sleep to baseline (24 h before social defeat) using two parameters that indicate sleep quality, EEG power and sleep duration. NREM sleep after SDS showed a significant decrease in EEG power in the delta band (Fig. 3A) and a shortened duration (baseline NREM sleep, 81.1 ± 5.1 min; NREM sleep after SDS, 40.8 ± 4.8 min, Fig. 3B). In addition, we measured the latency to the first NREM episode after SDS. Intruder mice remained awake for 27.2 ± 3.4 min, after which NREM sleep started to increase (Fig. 3C). These results suggested that acute SDS results in increased NREM sleep with poor sleep quality, shorter episodes, and longer latency.

Although the total time of REM sleep in the first 3 h after acute SDS did not significantly increase, it showed a robust increase at one time point (Fig. 2G–H). Unlike NREM sleep, the first increase in REM sleep had EEG power, especially theta power, which is a prominent feature of REM sleep in rodents [24], similar to baseline REM sleep (Fig. 3D). In addition, there was no difference between the episode duration of the increased REM sleep and that of normal REM sleep measured one day before SDS (Fig. 3E).

### Anxiolytic Effect of Increased NREM Sleep after SDS

**Fig. 4:**
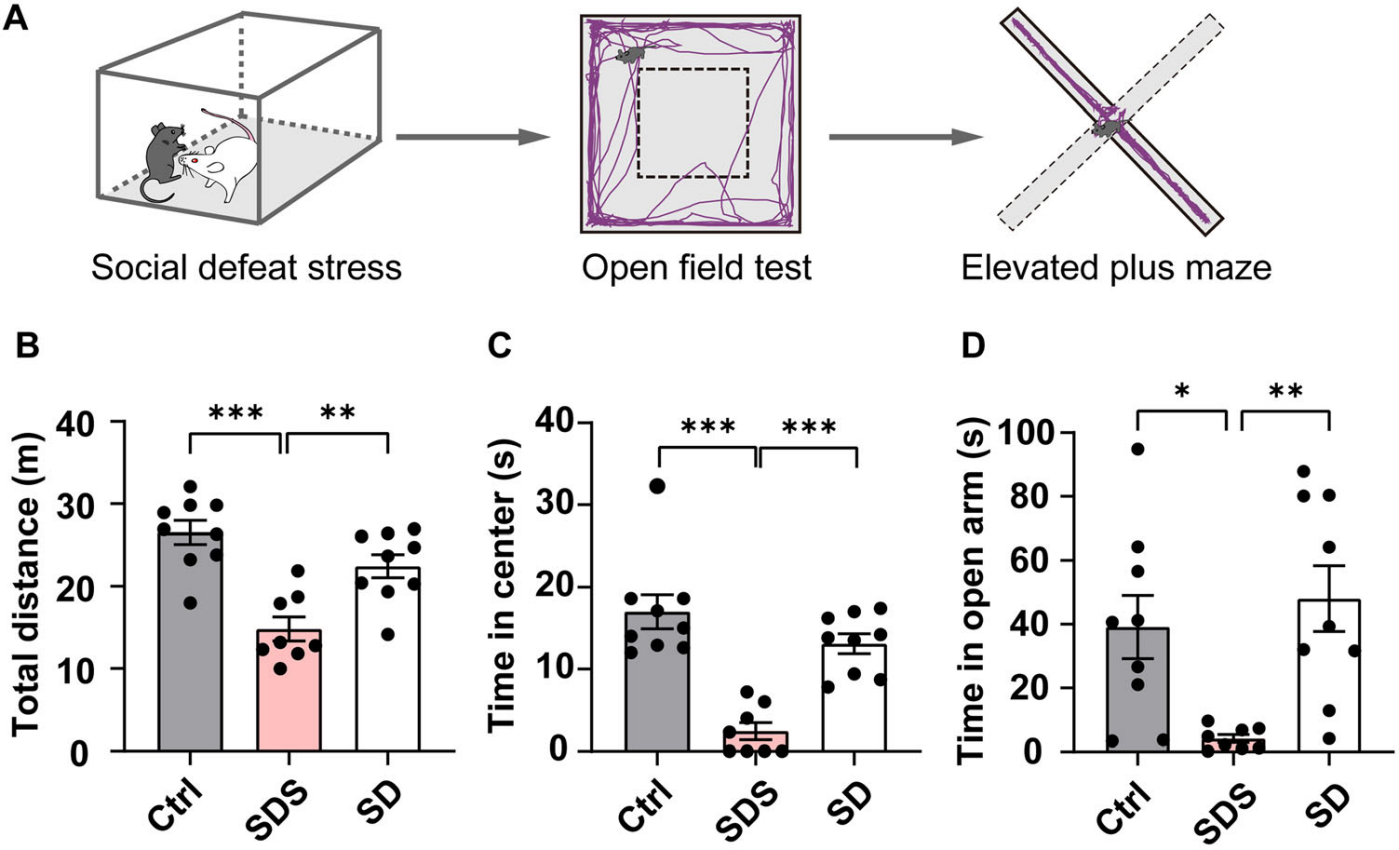
Increased anxiety-like behaviors following acute SDS. **A** Behavioral paradigms of anxiety-like behavioral tests immediately after SDS. After 10 min of SDS in the cage of a CD-1 mouse, the intruder mouse is tested in the OFT and EPM for 5 min. Purple traces, representative activity of the defeated mouse in the OFT and EPM after SDS. **B**–**D** Total distance travelled during 5 min in the OFT, and the time spent in the center of the OFT and open arms of the EPM, of control mice (Ctrl), SDS mice, and control mice with sleep deprivation (SD). **P < 0.01, ***P < 0.001, one-way ANOVA with Tukey’s multiple comparisons; *n* = 8–9/group.

Several studies have demonstrated that sleep loss is closely associated with the occurrence of symptoms of anxiety [18, 19, 24, 25]. To investigate the interesting hypothesis that the NREM sleep after social defeat participates in the regulation of anxiety, we first assessed the anxiogenic effect of acute SDS in mice in the active period. After being placed in the home cage of a CD-1 mouse, the intruder mouse suffered defeat for 10 min (Fig. 4A). Immediately afterwards, we tested the anxiety-like behaviors using the OFT and EPM (Fig. 4A). Compared with control mice that never suffered defeat, those exposed to social defeat showed decreased activity in the open field (Fig. 4B). The anxiety-related criteria, time in the center of the OFT and open arm of the EPM, were both markedly decreased (Fig. 4C, D; total distance: Ctrl, 26.5 ± 1.4 m; SDS, 14.8 ± 1.5 m, 44.1% reduction; OFT: Ctrl, 17.0 ± 4.1 s; SDS, 2.4 ± 1.0 s, 85.7% reduction; EPM: Ctrl, 39.1 ± 0.9 s; SDS, 4.1 ± 9.2 s, 89.4% reduction). These results demonstrated the anxiogenic effect of acute SDS.

Because sleep deprivation is considered to be an anxiogenic factor in humans, we investigated whether acute sleep deprivation with gentle touch for 3 h causes anxiety-like behaviors in normal mice. In the absence of SDS, 3 h of sleep deprivation changed neither locomotion (Fig. 4B), nor anxiety-like behaviors in the OFT and EPM in control mice (Fig. 4C–D).

**Fig. 5:**
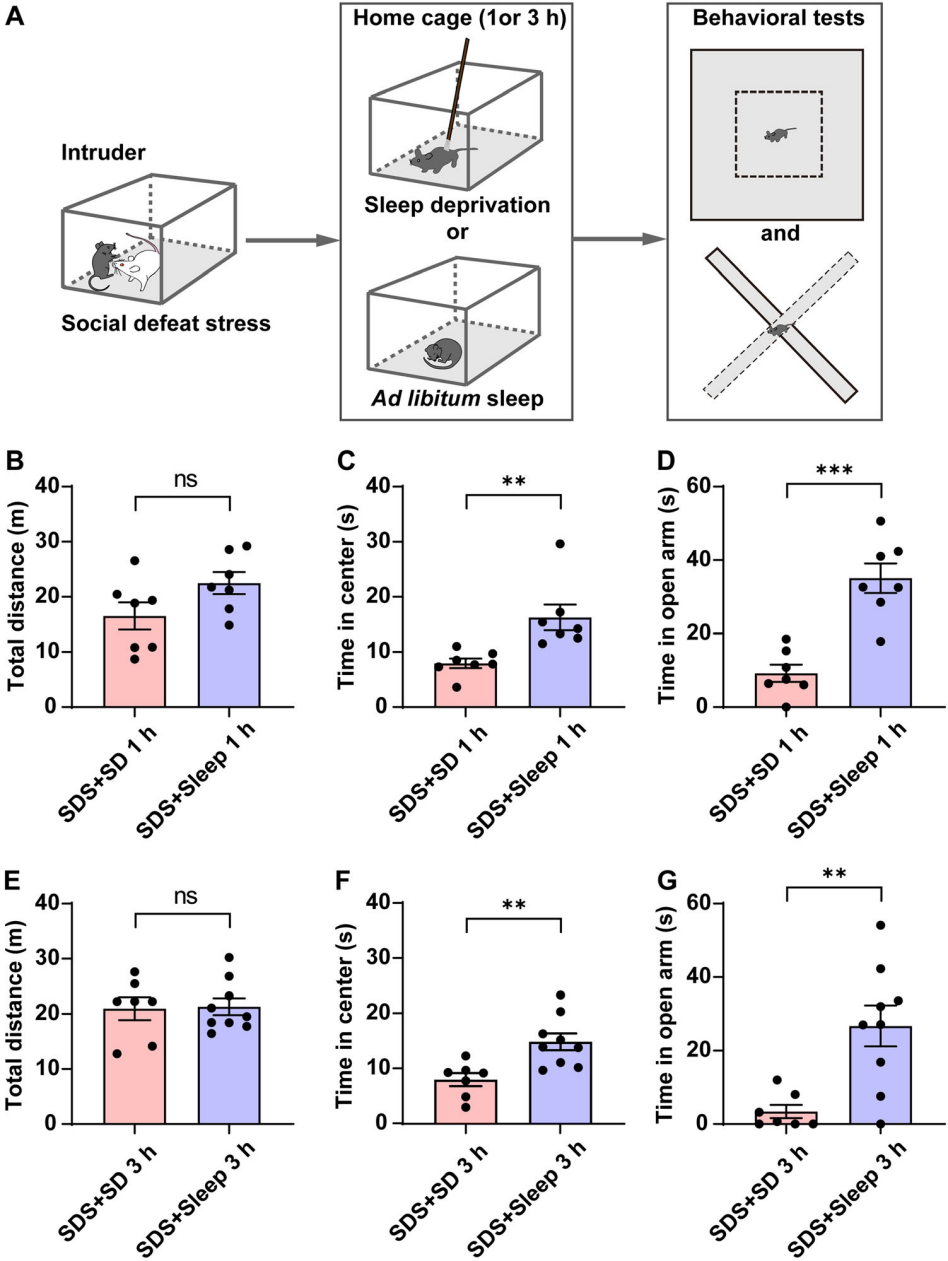
The anxiolytic effect of increased NREM sleep after SDS. **A** Experimental design of sleep deprivation and behavioral tests. After SDS, the intruder mouse is returned to its home cage for 1 h or 3 h, one group for *ad libitum* sleep (SDS + sleep 1 h or SDS + sleep 3 h) while the other is sleep-deprived (SDS + SD 1 h or SDS + SD 3 h). Each mouse was tested in the OFT and EPM 1 h or 3 h later. **B, E** Total distance travelled during 5 min in the OFT. **C, F** Time spent in the center of the OFT. **D, G** Time spent in the open arms of the EPM. **P < 0.01, ***P < 0.001, paired two-tailed Student’s *t*-test; *n* = 7–9 mice/group.

To assess the role of increased NREM sleep in modulating anxiety, the defeated mice were divided into 2 groups: mice with *ad libitum* sleep in their home cage, and sleep-deprived mice. After 1 h or 3 h of *ad libitum* sleep or sleep deprivation after SDS, each mouse was tested in the OFT and EPM (Fig. 5A). No significant locomotor changes were found between these groups (Fig. 5B, E). Compared with mice sleep-deprived for 1 h or 3 h, those with the same amounts of *ad libitum* sleep showed increased time in the center of the OFT (Fig. 5C, F) and open arms of the EPM (Fig. 5D, G). These results provide direct evidence for an anxiolytic role of increased NREM sleep after SDS.

**Fig. 6:**
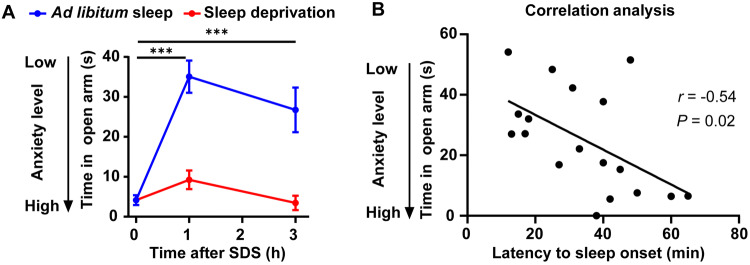
Relationship of sleep and anxiety-like behaviors after acute SDS. **A** Anxiety level (time spent in open arms of the EPM) decreases quickly with increased sleep after SDS, compared with sleep-deprived mice (***P < 0.001, two-way ANOVA with Tukey’s multiple comparisons). **B** Correlation analysis of the anxiety level against latency to sleep onset after acute SDS (fitting equation, Y = −0.5757*X + 44.88; Pearson’s *r* = −0.54).

To exclude the influence of extended wakefulness caused by sleep deprivation on anxiety-like behaviors, we compared the anxiety-like behaviors of the two groups with sleep deprivation for either 1 h or 3 h. No significant difference was found (Fig. 6A). Similarly, there was no significant difference between the groups with *ad libitum* sleep for either 1 h or 3 h; both showed decreased anxiety levels after SDS (Fig. 6A). Taken together, these results demonstrated that sleep is necessary to reduce anxiety-like behaviors. Within the 3 h after social defeat when NREM sleep increased, the anxiety-level did not depend on the length of sleep because sleep *ad libitum* for 1 h and 3 h had similar effects on anxiety-like behaviors, and 1 h of sleep after stress is sufficient to relieve anxiety.

In addition, we found that the latency to sleep onset reflected the anxiety level after SDS and natural sleep, as it was positively correlated with anxiety-like behaviors (or a negative correlation with time in the open arms) (Fig. 6B; *r* = −0.54, 95% confidence interval = [−0.80, −0.10], *P* = 0.02). Supporting the hypothesis that more anxious individuals find it harder to fall asleep, the latency to sleep onset following acute SDS predicted the level of anxiety: mice taking more time to fall asleep were more likely to spend less time in the open arms of the EPM after 3 h of *ad libitum* sleep; this is interpreted as indicating a high level of anxiety.

All the above data suggest the essential role of sleep in the relief of stress and modulation of anxiety.

## Discussion

In this study, we focused on the anxiolytic effect of increased NREM sleep in mice experiencing acute social defeat. Our results showed that acute stress in the active period increased the duration of subsequent NREM and REM sleep. In particular, NREM sleep but not REM sleep significantly increased within the first 3 h after social defeat. Deprivation of this increased NREM sleep maintained a high level of anxiety in socially defeated mice, but sleeping *ad libitum* returned the anxiety level to normal.

The strong increase in sleep after acute social defeat indicated that sleep may function to offset the mental load imposed on the nervous system during wakefulness. In fact, social conflict in rodents enhances the amount and intensity of subsequent sleep following stress-induced insomnia [8]; sleep regulation seems to be a natural means of recovery from stress.

Usually, the anxious state induced by acute social defeat does not last long in naive mice. Mice rapidly recover to a normal anxiety level, which prevents the pathogenesis of anxiety disorders. However, the mechanism by which individuals spontaneously recover from stress is still not well studied [5]. Here, based on our data, we found sleep to play an essential role in the stress recovery process. We believe that if acute stress is not alleviated through sleep, it is likely to develop into an emotional disorder such as anxiety disorder or depression. In addition, NREM sleep has a faster response than REM sleep to acute stress. On the other hand, clinical studies show that the percentage of NREM sleep is decreased in individuals with high anxiety [26], suggesting that NREM sleep is essential for resisting the development of anxiety disorders.

In laboratory studies, anxiety or depression is often modeled with chronic social defeat for 7 days or 10 days. In chronic social defeat, the presence of sensory contact with a CD-1 mouse may change the subsequent sleep profile, which would have profound effects on recovery from anxiety. During the sensory contact period, the intruder mouse remains in the aggressor’s home cage but separated from it by a baffle that allows visual, auditory, and olfactory interaction with the aggressive CD-1 mouse [23, 27, 28]. For example, one study with a 20-min period of sensory contact with a CD-1 mouse after 5 min of social defeat interaction showed that wakefulness is increased in the first 3 h followed by a later increase in sleep [13]. This result provides clues to explain why the anxiolytic effect is absent in the chronic SDS model in which the sensory contact lasts for 24 h.

Here, we used sleep deprivation to study the function of increased NREM sleep in anxiety. Indeed, chronic sleep deprivation is reported to be closely associated with anxiety and negative emotion in humans [18, 29–31]. Anxiety-like behaviors can be induced by sleep deprivation for 72 h but not 24 h in mice [19]. However, the reports about the effect of sleep deprivation on anxiety are inconsistent, as some animal studies point towards an anxiolytic-like effect of sleep deprivation [32, 33]. Here, we demonstrate the anti-anxiety and mood-regulating effects of sleep after acute SDS, using short-term (3 h) sleep deprivation which did not increase anxiety (Fig. 4B–D).

In our results, REM sleep-rebound occurred later than the increased NREM sleep. REM sleep is an adaptive response to stressful situations [34] and plays a functional role in the regulation of emotions [25, 35]. It will be important for future studies to further elucidate whether and how REM sleep interacts with stress-induced anxiety-like behaviors.

However, the mechanism underlying the anxiolytic effect of sleep is still unclear. The reduction of anxiety-like behaviors is unlikely to be related to memory loss because NREM sleep contributes to memory consolidation [36–38]. We speculate that activation of anti-anxiety circuits, inhibition of anxiety-related circuits, or release of anti-anxiety substances may occur during sleep. The effect of sleep, including NREM sleep and REM sleep, on the stress-induced changes in neuronal plasticity is of great significance [39, 40]. Further study is needed to reveal the mechanism underlying the anxiolytic effect of sleep. In conclusion, our findings suggest that NREM sleep after stress is important for emotion regulation and is a potential means of relieving or even treating anxiety.
